# Challenges and considerations in determining the quality of electronic performance & tracking systems for team sports

**DOI:** 10.3389/fspor.2023.1266522

**Published:** 2023-12-20

**Authors:** Sam Robertson, Grant Malcolm Duthie, Kevin Ball, Bartholomew Spencer, Fabio Rubens Serpiello, Jade Haycraft, Nicolas Evans, Johsan Billingham, Robert James Aughey

**Affiliations:** ^1^ Institute for Health & Sport, Victoria University, Melbourne, VIC, Australia; ^2^ School of Exercise Science, Australian Catholic University, Sydney, NSW, Australia; ^3^ Fédération Internationale de Football Association (FIFA), Zurich, Switzerland

**Keywords:** GPS, optical, football, soccer, tracking

## Abstract

Electronic performance & tracking systems (EPTS) are commonly used to track the location and velocity of athletes in many team sports. A range of associated applications using the derived data exist, such as assessment of athlete characteristics, informing training design, assisting match adjudication and providing fan insights for broadcast. Consequently the quality of such systems is of importance to a range of stakeholders. The influence of both systematic and methodological factors such as hardware, software settings, sample rate and filtering on this resulting quality is non-trivial. Highlighting these allows for the user to understand their strengths and limitations in various decision-making processes, as well as identify areas for research and development. In this paper, a number of challenges and considerations relating to the determination of EPTS validity for team sport are outlined and discussed. The aim of this paper is to draw attention of these factors to both researchers and practitioners looking to inform their decision-making in the EPTS area. Addressing some of the posited considerations in future work may represent best practice; others may require further investigation, have multiple potential solutions or currently be intractable.

## Introduction

Electronic performance & tracking systems (EPTS), for example Global Navigation Satellite Systems, Local Positioning Systems or optical systems are commonly used to track the location and velocity of athletes in many sports, along with various associated instruments such as balls and racquets. Consequently, EPTS incorporating a range of technologies experience widespread applications in many sports. These applications include the assessment of physical characteristics and tactical behaviour of athletes, players or teams in competition (1–4). They are also used to help design training to replicate perceived key features of these competitive environments (5, 6) and inform monitoring of athlete workloads (7–9). More recently, EPTS have also been used to help with adjudication of many sports such as football for purposes such as reviewing offside and goal line decisions (10). Output from EPTS is also increasingly being utilised for broadcast and digital media purposes to engage sports fans and consumers (11).

The accuracy and reliability of EPTS is of practical importance to all of the above stakeholders, although the relative importance for each may vary depending on their utility. For instance, adjudication of an offside call in a football match requires centimetre-precise accuracy with respect to tracking the position of multiple players, along with the ball. The development of a heat map visualisation showing the main areas of a football pitch that a player inhabits for fan engagement purposes may be less crucial. Nonetheless, as technology improves and stakeholder financial outlay and expectations increase, an understanding of the systematic, random and methodological factors that have the potential to influence the output of an EPTS system too become increasingly of use.

The influence of systematic, such as the type of EPTS, or number of cameras and methodological, such as filtering applied to positional or speed data, factors on EPTS validity is non-trivial and explored in detail below. This manuscript aims to build on existing work examining these factors in team sports; see (2). It is plausible that differences between different EPTS could be predominantly attributable to these factors and less to do with the “true” accuracy of the system itself. For instance, two different EPTS may have a theoretically equal level of accuracy, however may display different results due simply to differing set ups, filtering or through tracking different parts of an object of interest. When one of the systems is considered a benchmark or gold standard, this can lead to incorrect conclusions being drawn on the quality of a system, thus resulting in a potential negative outcome for a manufacturer. As the tracking of humans and objects in sport becomes increasingly detailed, along with generalised improvements to commercial offerings and varied hardware, questioning as to what constitutes a gold standard for different purposes may result.

In this manuscript a number of challenges and considerations relating to the determination of EPTS validity for team sport are outlined and discussed. The aim of this paper is to draw the attention of both researchers and practitioners to these areas when looking to inform their decision-making around EPTS. Addressing some of the posited considerations in future work may simply represent best practice, whereas others may require further investigation, have multiple potential solutions or currently be intractable.

## Systemic considerations

Three main types of EPTS exist in the commercial market. These are Global Navigation Satellite Systems (GNSS), local positioning systems (LPS) and video-based (optical) systems. Light detection and ranging systems (LiDAR) are a newer type of system experiencing some popularity (see 12, 13), however remain less commonly used comparatively. It is likely that further methodologies will emerge in the coming years. Some considerations on the selection of these systems, how their accuracy may be influenced and considerations for test setup are briefly discussed below.

### Global navigation satellite systems (GNSS-based)

EPTS utilising Global Navigation Satellite Systems (GNSS) (14) rely on the precise measurement of time from an atomic clock on each satellite for the calculation of the length of time it takes a radio signal to travel from the satellite to the GNSS receiver on earth. Whilst theoretically only four satellites are needed to obtain an accurate determination of position of the receiver on Earth (15), it is also the relative location and spread of those satellites that influences accuracy. It is typically true, however, that reception from more satellites is better than fewer. For example, GPS-derived velocity from three satellites had an approximately double higher mean error compared to six satellites during cycling (16). The improved accuracy with a greater number of satellites can be quantified as a reduction of the position dilution of precision (in particular the horizontal dilution of precision, HDOP) (17). Therefore the HDOP should be both measured and reported when establishing the accuracy of GNSS (18).

Most GNSS systems used in sports currently employ multiple satellite constellations such as the GPS (USA), GLONASS (Russian Federation), Beidou (PRC) and Galileo (European Union). Whilst it is intuitive that multiple constellations would increase the number of satellites for communication with a receiver at a given location (19) this is only true if one constellation could “fill gaps” in coverage from another (20). For example, when a device integrating GPS, GLONASS, BeiDou and Galileo was compared to a GPS-only device, the static 3D position accuracy was improved 33.8%–38.5%, while dynamic accuracy by 12.2%–39.8% (20). The magnitude of improvement in accuracy with additional satellite constellation access points to a greater number of satellites being better, but does not inform on the minimum number of satellites required for acceptable accuracy. Future research may address that question, but in order to do so will require being able to add signal from satellites and quantify the enhancement of accuracy. There is also possibly a saturation point where more satellites does not equate to any further improvement in position accuracy.

When selecting an appropriate environment or venue for GNSS validation testing against another system or a gold standard, it is important to note that the accuracy of GNSS is directly affected by the unit's vicinity to buildings that obstruct the view of the sky (2). The horizontal precision of dilution is greatly improved when a GNSS sensor's validity is assessed in the centre of a sporting oval compared to the edge, near a single-storey stadium stand (21). However, a device designed for use in the sporting environment must be able to operate accurately with limited access to the sky typical of sporting arenas globally, with the exception being indoor stadiums.

### Local positioning systems (LPS-based)

Tracking systems based on local positioning systems (LPS), typically measure the position of a moving object or person by analysing the time and/or angle of arrival of a signal between fixed anchors and a receiving unit (22). The position calculation in LPS is similar to the methods used in GNSS, as there are satellites with known positions and a receiver with an unknown position and a time offset due to a missing synchronization between the receiver and satellites (23). Early LPS tended to use radio frequency communication between anchors and receivers (24), however this technology was susceptible to multipath fading (25), and newer systems tend to use Ultra Wideband technology to overcome this deficency (26).

Similar to GNSS where an increased number of satellites may equate to increased accuracy of a system, it may be assumed that a greater number of anchors in LPS would lead to a greater accuracy of the system. However, again similar to GNSS, accuracy is not necessarily enhanced solely through an increase in anchors as this also reduces the available bandwidth and therefore sample rate for measurement of activity (27). A reduced sample rate may lead to short, rapid movements commonly seen in sport being poorly captured. An increase in anchor numbers may also complicate the synchronisation of signals, critical to calculate time of arrival of a signal in an LPS (27, 28). It is highly likely that the optimal number of anchors is situation dependent. For example, in a simulation project with a 30 m × 30 m capture area, the mean squared error of positioning (∼0.5 m with three anchors) stabilised when 8–10 anchors were used to ∼0.1 m (29).

### Optical-based

Optical based player tracking initially constituted a manual or semi-automated process that required labour-intensive human intervention (30). Typically, matchplay was recorded and a notational analysis process employed (for a review of notational analysis, see 31). Advances in both computer vision hardware and analytical processes have further automated the optical tracking process.

Computer vision applications in sport range from single (32), multiple camera set-ups (33, 34) or panning cameras such as those used in television broadcast (11). Multiple camera systems are commonly superior to single-camera setups in terms of accuracy, due to an effective increase in resolution (each camera focusses on a smaller area of the pitch, increasing pixel per metre ratio) and a greater ability of multiple cameras to deal with occlusion of players common in team sport (35–37). In multiple camera systems it is also possible to switch view for tracking from an occluded camera to one with a better view (38).

The accuracy of an optical tracking system is not merely related to how many cameras it incorporates. The setup of cameras in a given stadium can also greatly affect the accuracy of a system. The height of cameras needs to be sufficient to ensure as uninterrupted a view of players as possible (34, 39). A camera angle of between 10 and 20 degrees from the pitch has been recommended (40). There is the possibility of a trade-off between the installation height of cameras and the capacity of those cameras to ensure clarity of image is retained with a greater distance from the pitch. Further, players in the foreground of an image may be orders of magnitude “taller” for pixel count than players in the background (41). Lower camera positions will also reduce horizontal plane resolution for the axis aligned vertically on the screen compared to horizontally.

The use of high definition video is ubuiquitous in optical tracking in sport, and this high definition allows for robust detection of players (42). In addition to a high definition image, cameras need to have an appropriate shutter speed, frame rate, focal length of lens, and sensor (as a mechanism for capture in the camera) to capture key movements of players and or the ball during competition (40). This is an ongoing area of refinement for many commercial providers and no specific recommendations are provided here.

Camera calibration in optical tracking involves a process whereby the video image is scaled and anchored to the two or three-dimensional playing pitch area to enable the calculation of *x*, *y* and potentially *z* location and speed of players and/or the ball (43). Scaling ensures distances and speeds are appropriately measured while anchoring provides a reference to enable player position on the pitch to be identified. This calibration is critical in ensuring the relative location of players is correctly obtained, especially when multi-view images are obtained from multiple camera systems (44). Techniques for calibration range from the use of known positions within a field of play—for example line markings on a pitch (45) through to simulation models (46).

The above paragraphs describe key elements in the capturing of the video image, the next stage for optical systems is the identification and subsequent tracking of players. A variety of methods have been applied to identify and subsequently track players, and a review of these is beyond the scope of this article. Briefly, early systems identified players by first subtracting the background (47), and then creating shape-specific occupancy maps (42), but these methods only identify isolated players (48). In theory, background subtraction methods should be stable in varying light conditions (42), but for validity testing lighting should be as stable as possible. The use of convolutional neural networks for identification and tracking has grown in popularity in recent times to overcome some of the shortcomings of earlier methods, and greatly decrease the processing time required for tracking (48, 49).

### Which part of the athlete, ball or team is being tracked?

Another fundamental difference between various types of EPTS relate to which part(s) of an object are actually tracked. For athlete tracking, GNSS- and LPS-based EPTS typically utilise sensors that are housed within a specially-designed garment or jersey, with the the device located between the scapulae (e.g., 26). Optical tracking systems differ in that they tend to track objects via image segmentation through various means of image recognition (50). A common method is through establishing a rectangle or cylinder, which, using an estimate of the mass of the object's parts then identifies its centre (50). These optical tracking systems tend to locate a position near the centre of the pelvis of the player (51).

Previous work has shown that these seemingly minor differences between methodologies have the potential to influence the output (52, 53). Linke et al, 2019 (52) compared the centre of the scapulae (approximating the location of a GPS or LPS unit) with the centre of the pelvis (approximating an optical system) finding that the magnitude of the differences were dependent on the underlying movement characteristics. Only small differences existed at lower running speeds but these increased at higher speeds. These were also more pronounced where greater acceleraton events existed (e.g., acceleration/deceleration, change of direction). This suggests that the differences between shoulder-mounted and optical systems can be at least partially explained by upper trunk motion. For example, during an acceleration, a player will lean forward, positioning the shoulders ahead of the hips, while the opposite occurs during deceleration.

The consideration as to which part of the body should be identified is an important factor in player tracking. Linke et al., 2018 (53) suggest that under the assumption the tracking system is attempting to locate the body as a whole, centre of mass (COM) should be considered as a valid criterion. Further, they argue the solution should be a biomechanical one and not prescribed by the location a particular system tracks. The concept of using COM is justifiable, given it is the point that represents body movement as a result of all forces applied to, or by, the body and has direct relevance in balance and jumping tasks. However it should be noted that tracking different body locations is likely to also provide varying data on usual parameters of interest including position, speed and acceleration as well as potentially in tactical movement pattern analysis. The extent to which these differences may influence practice requires further investigation. It is also worth noting that the use case for the data should also be front of mind when considering decision making in this area. For instance, a practitioner wishing to use the data for tactical purposes may prefer a different solution to one looking to accurately determine the lower body acceleration characteristics of an athlete.

**Figure 1:**
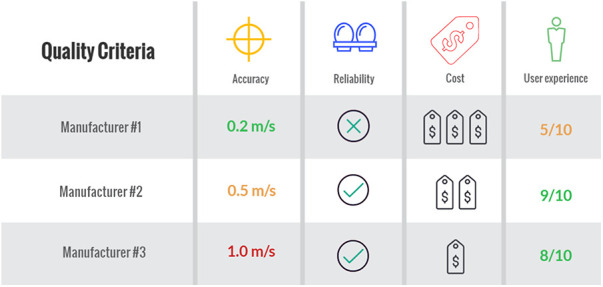
Example decision-making checklist whereby a stakeholder can offset different features of an EPTS and compare between systems prior to acquisition.

Determining the interchangeability between the three above system types is important as many athletes and teams may be exposed to one type of system in training and another in competition (5). Further, with the rapidity of technology and hardware developments, systems which athletes and teams are exposed to have the potential to change on a regular basis. When comparing one system to another, a number of contextual features require consideration rather than solely accuracy and reliability. As an example, changes to the configuration of a system can in turn influence both their feasibility and cost. For instance, a 16 camera system is expected to produce more accurate tracking comparative to one that utilises only two, however assuming a higher cost, a question may emerge with respect to how much better it should be in order for it to be considered a better investment? Further, an LPS system incorporating portable nodes might improve the quality of capture relative to GNSS, but set up and pack down time might render it infeasible in some scenarios. Figure 1 provides an example of how certain features of a system may be compared against each other based on the needs and budget of a given organisation.

## Methodological considerations

Comparison of data from each of the different abovementioned systems is only part of the story in determining the quality of an EPTS. A range of other considerations under the control of the human user or relating to the specific use case also exist; these can be defined as methodological rather than systematic. In particular factors relating to data collection, processing and analysis methodology all have the potential to meaningfully influence the EPTS output and some of these specific considerations are expanded upon below.

### Limitations of gold standard comparison in the field

One of the biggest challenges in evaluating the quality of EPTS is the lack of a ground truth by which to compare. This is not only in terms of measurement hardware, but also in terms of methodology. With respect to the former, three-dimensional (3D) motion capture systems are considered as a gold standard for the measurement of kinematic data (54). Appropriately set up and calibrated, these systems have been reported to provide sub millimetre precision in measurement (53).

**Figure 2:**
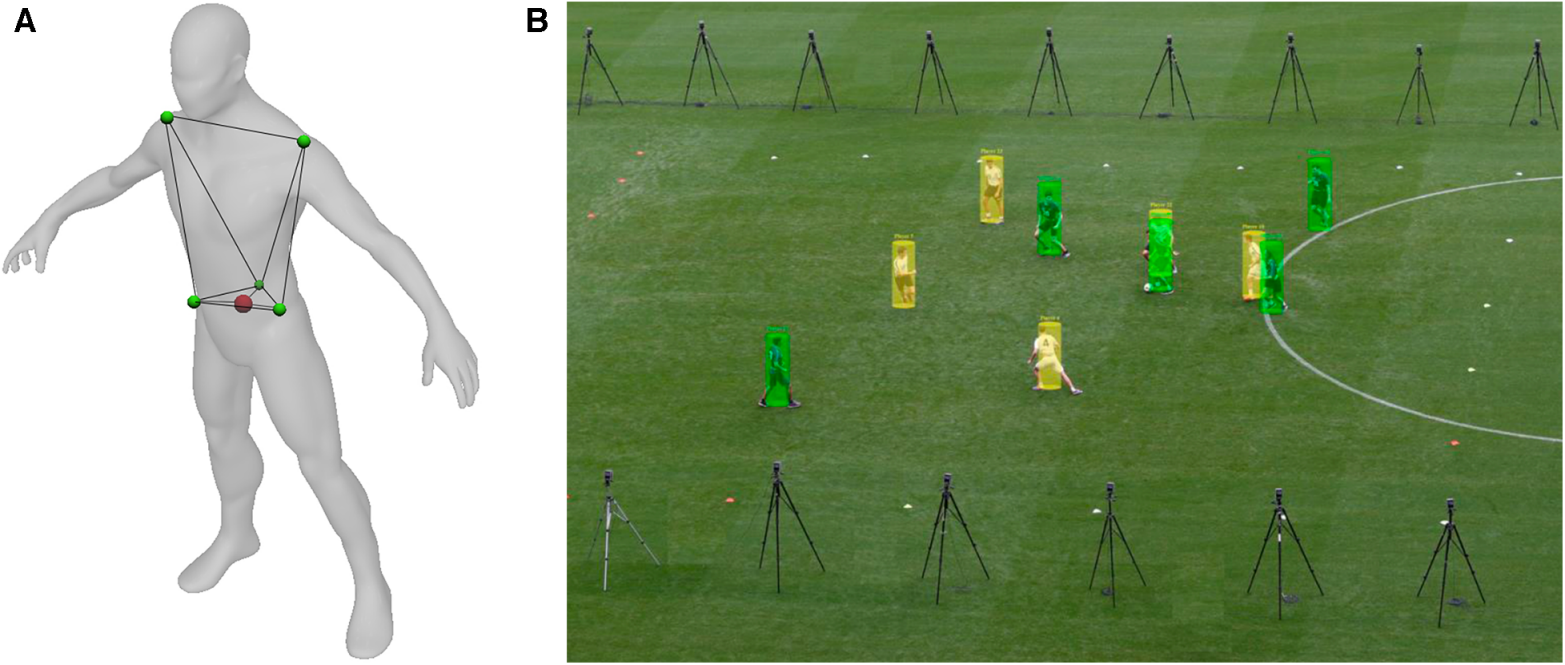
Differences in the typical manner by which EPTS track objects; in this example, a human. In (**A**) the GNSS received is located between the scapulae of the individual, (**B**) shows the method typically used by optical-based systems and (**C**) infers centre of mass of an object via a 3D marker system.

These 3D motion capture systems track differently to EPTS in the ways that are described above to measure a specific body location or approximate COM. In a laboratory setting, this is typically performed using the segmental method with markers placed on the trunk, upper and lower body to calculate COM (e.g., 55). While the use of all segments will provide the most comprehensive methd of estimating COM location, it is a lengthy process and consequently there have been attempts to find COM with fewer datapoints. Saini et al., 1998 (56), for example, reported that COM could be reasonably predicted during gait by tracking the pelvis only. Although the rigour around this approach requires further clarification, this same method has been used in player tracking studies to monitor COM movement in football-specific movements (e.g., 53; Figure 2).

Further, current limitations to 3D motion capture hardware prevent it from being utilised in the large spaces often present in most sports. Consequently, comparison with EPTS *in situ* competition environments is normally not possible, although improvements to these systems may mean it becomes a reality in the future. Thus, reconstructed circuits or small-side games (SSG) have often been used instead (i.e., 57, 58). Despite the obvious practical benefits, unfortunately, a number of disadvantages of this approach may apply. For instance, it may lead to the movement profiles of tested participants being different in testing; most notably they may not reach the peak of velocities in the capture space that they may record in competition. This requires a different approach, such as having players start running outside of the test area so they have sufficient distance to reach close to top speed by the time they enter the test area.

With all of these considerations in mind, the terms “gold-standard” or “criterion” often used with respect to 3D motion capture systems, may not be appropriate for the purposes of EPTS testing (particularly outdoors in the field). It is recommended that 3D motion capture systems are referred to as establishing concurrent validity rather than criterion validity or a gold standard, as per the terminology outlined in (59), and be used for validation of EPTS.

### How much data is needed when evaluating an EPTS?

With the previous section in mind, a practical question emerges with respect to how much data is required in order to make an informed evaluation on an EPTS. Small samples may mean that random variation can play a part in any between system differences that are reported. For example, high velocities are harder to recreate in testing environment and thus harder to obtain sufficient data on, due to size limitations of the capture space and the difficulties in participants repeatedly reaching these speeds. It is also important to understand that participants are not robots either—repeat test sessions using the same participants can render them tired later in the day, thus causing lower than competition-level velocities. This tiredness in later tests may mean that when multiple manufacturers are being assessed, those tested earlier in a day may be subject to unfair disadvantage (assuming higher velocities are more difficult for most EPTS to accurately track). Other somewhat uncontrollable factors may also be influential—a change in surface and weather conditions during the test session has the potential to alter the velocities and movement displayed by athletes in testing as well. This may be more problematic for some types of EPTS more than others (i.e., an optical-based system may struggle with changes to sunlight more so than GNSS-based).

**Figure 3:**
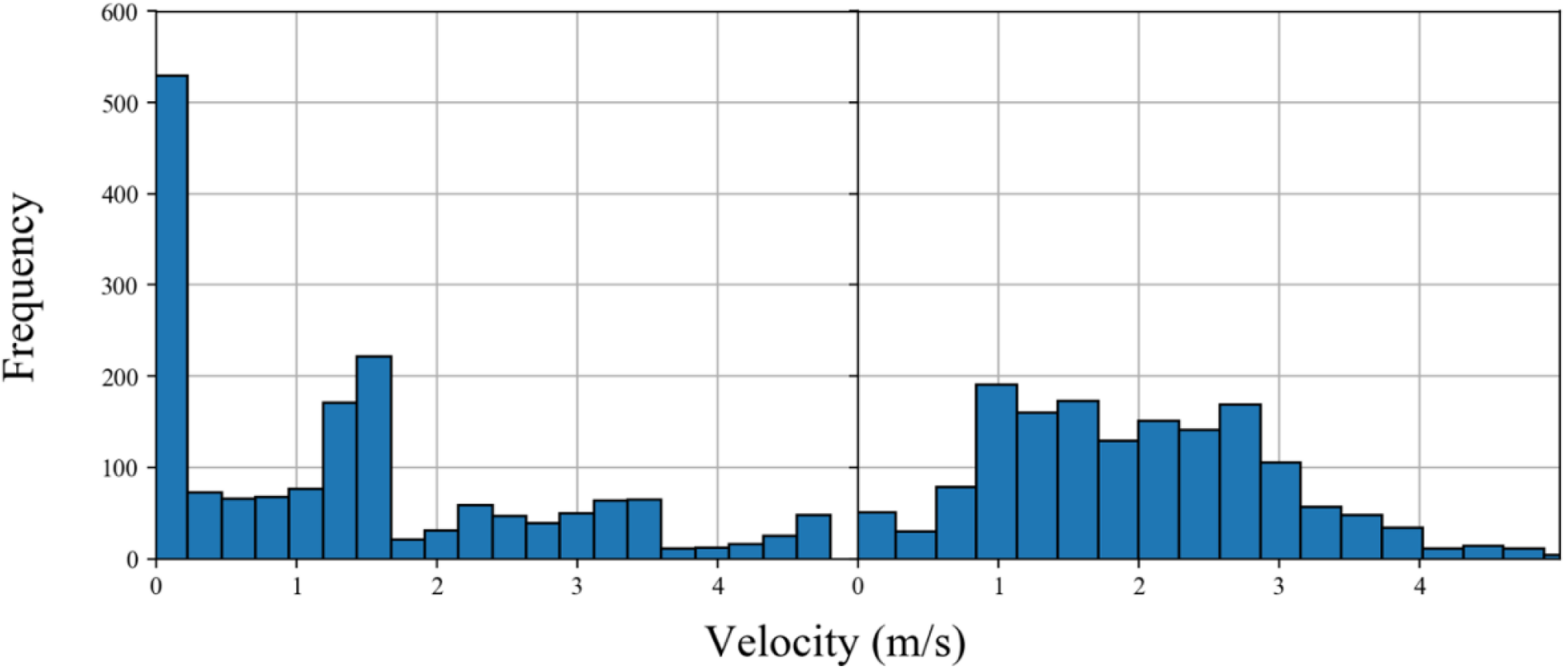
Distribution of velocity of an exemplar athlete in (**A**) testing and (**B**) competition. Clear differences in the number of samples (frequency on the *y*-axis) recorded at lower velocities are observed, thus questioning the representativeness of the testing environment and generalisability of the findings.

Two main considerations exist for researchers when deciding on how much data is required to gain a reasonable representation of EPTS validity. The first relates to the extent to which the test conditions resemble competition. The second relates to the point at which a realistic representation of the EPTS error is reliably acquired. Relating to the design of the test session, the former is typically defined as a measure of the “representativeness” of a test (60). One of the ways in which this can be assessed is through inspection of the distribution of velocities (Figure 3). Figure 3A shows the typical velocity profile of a player during competition, whereas Figure 3B shows their output in a testing event. Clearly, more time is spent standing still by the test participant in the second example, thus if an EPTS found it harder to track a participant in this condition comparative to say, walking, then they may be being unfairly evaluated with respect to the typical conditions they would expect to perform to in competition.

**Figure 4:**
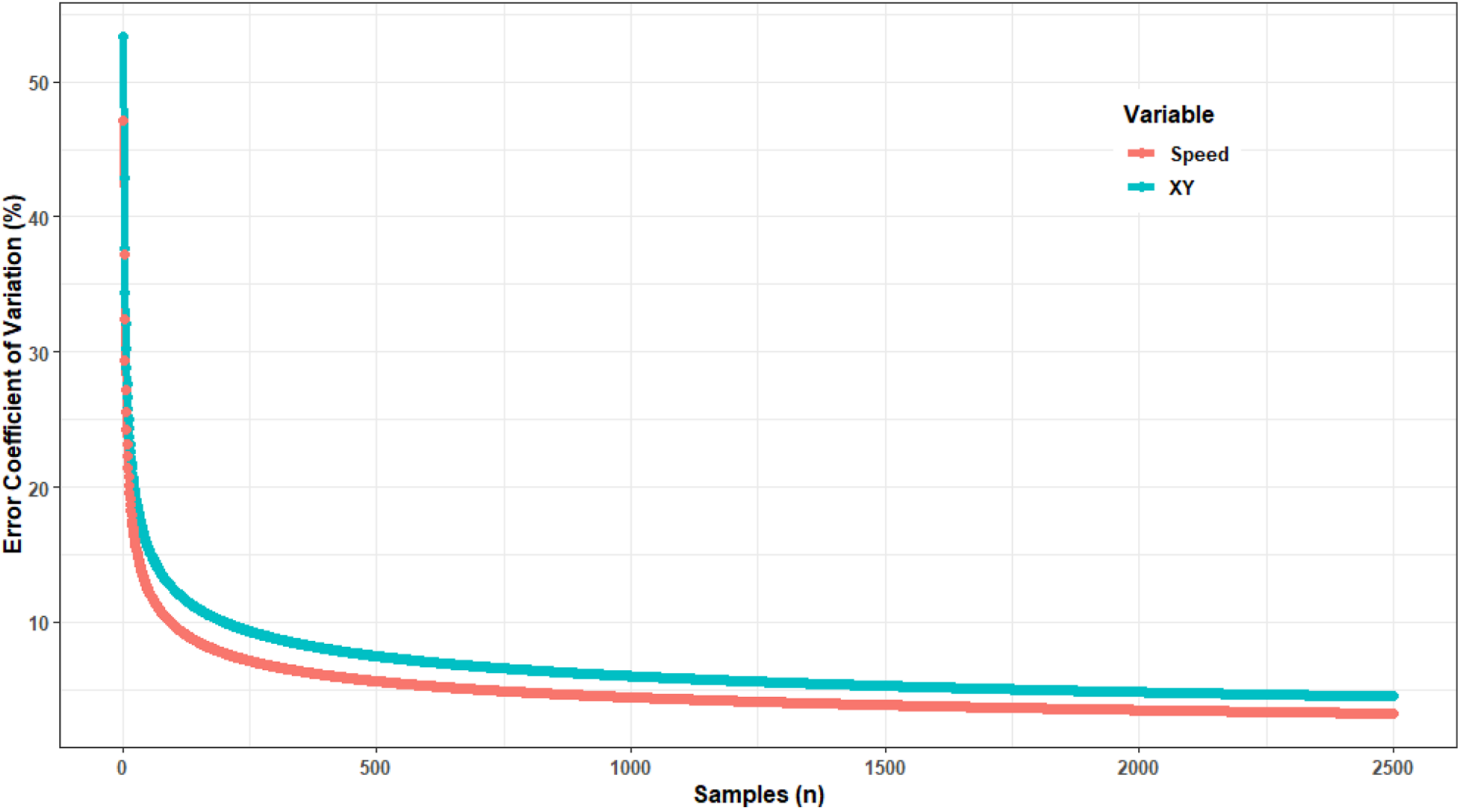
Change in differences between two EPTS systems (expressed as a coefficient of variation) as a function of number of samples. A flattening of the curve (stabilisation of the difference) can be used to inform the number of samples required when conducting comparisons.

There is a given time it takes for the differences between two systems to stabilise. Figure 4 shows an example whereby the more samples are collected, the differences (in this example, expressed as a coefficient of variation) of a system is reduced as more samples are obtained. The difference between the final error and the “cut point” on the plot can along with the information from Information obtained from Figure 4 could be used to inform the duration of a testing session. In this example based on actual data, it is clearly evident that some stabilisation is already present within 2,500 samples, which for a standard 10 Hz GNSS system is as little as 250 s (or just over 4 min of data). Mathematical techniques have also been employed to determine when the mean of a parameter or relationship stabilises (e.g., 61, 62). This exercise helps to make testing more efficient which is important when multiple teams need to be tested, saving pitch quality and grass, resulting in less analysis and ultimately resulting a less resource-intensive exercise to conduct. The authors recommend using a combination of the abovementioned approaches to inform the amount of data required.

### System synchronisation

**Figure 5:**
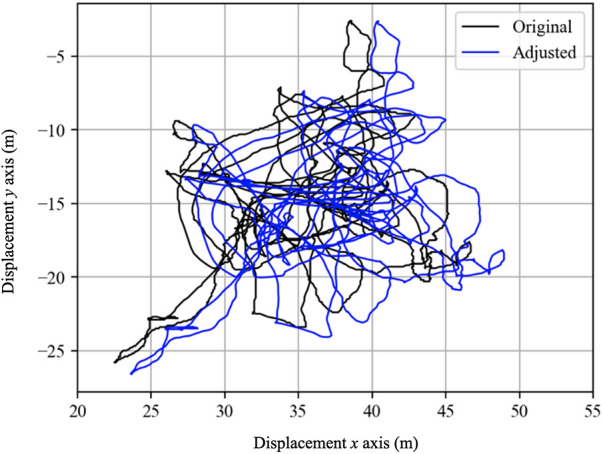
Example of two positional traces of athlete movement in a test setting. Although the traces are near identical, numerical differences would be reported due to systematic differences relating to synchronisation. In such instances, application of a cross-correlation and mean offset methodologies may present useful synchronisation approaches.

A number of considerations relating to synchronisation exist prior to comparing outputs between systems. One of the major difficulties in the assessment of position is that GNSS-baesd systems do not provide cartesian coordinates (*x*, *y* location on a pitch). Whilst it is possible to convert position as measured by GPS in latitude and longitude to Cartesian coordinates, there is potential for error in doing so (i.e., Figure 5). Specifically, global positioning systems communicate with satellites orbiting the earth. Each satellite contains an atomic clock allowing precise timing of a radio signal that is then detected by a receiver on earth. The distance to that receiver is then calculated by multiplying the transit time by the speed of light (299,792,458 m.s^−1^) (18). If a minimum of four satellites are in communication with the receiver, an accurate position can be triangulated via spherical trigonometry and expressed as latitude and longitude (63). Sources of potential error in converting the satellite signal into cartesian coordinates include the geodetic datum used (a coordinate system used to provide known locations) (64), the technique for computing latitude of which there are at least twenty available (64, 65), as well as the technique for calculating height (65). In addition to the method used for calculations, there is the possibility for error introduced by: satellite and receiver clock offset (66); ephemeris prediction error calculations made based on the health of the satellite and its current and predicted location (67); relativistic prediction error where the satellite and receiver are located at different gravitational potentials (66); atmospheric effects (68); and multipath and shadowing effects (66, 69). Collectively, the errors outlined above make it unreasonable to assess GNSS against a reference three-dimensional motion capture system for location, so validation should at this stage instead concentrate on velocity of movement.

Another typical consideration when comparing between system is frame rate. At this stage, most optical systems typically operate at 25 Hz (standard camera frame rate) while LPS can range from 10 Hz to 50 Hz. GNSS on the other hand (also often 10 Hz) provides both latitude and longitude (and height) from which speed can be derived. However, speed is more commonly established from the doppler shift method as this is suggested to be more accurate (70). The data collected by each system (XY for camera and LPS, Doppler Speed for GPS) is then used to calculate a large range of subsequent metrics (distance covered, speed, acceleration, power etc). Other related points worth briefly mentioning relate to the fact that most 3D motion capture systems used for comparison with EPTS sample data at a much higher rate. This means that typically some downsampling of this data is required, and in some instances also it may require upsampling of an EPTS system, which can ultimately lead to the creation of data points not directly measured by an EPTS provider. Such a process is typically undertaken using common methods such as linear interpolation (71). Neither approach is ideal, however constitutes a necessary step in order to facilitate the comparison.

### Treatment of “raw” data

It is unlikely that raw data is being made commercially available to end-users. Consequently, it is assumed that most EPTS manufacturers apply some form of filtering to their raw collected data, although specific details are often a well-guarded secret. Filtering is common practice in the handling of time series data in other pursuits outside of sport, such as atmospheric (72), stock market (73) and magnetic resonance imaging analysis (74). In many cases, both the tester and customer does not have access to what a manufacturer has used. This means that the preprocessing methods by companies are hard to reproduce, also making it difficult to compare directly with a criterion or concurrent measure whereby this information is known. Fortunately in some cases, for example in the use of EPTS for tactical purposes in football, opensource packages such as https://pypi.org/project/databallpy/ and https://floodlight.readthedocs.io/en/latest/ are starting to emerge, thus streamlining this process and facilitating comparisons.

In other cases however, trial and error with the exported data is typically required on behalf of the user in order to land on a format that shows the strongest agreement. Serpiello et al., 2017 (26) highlighted this issue where their initial analysis used a standard smoothing cut-off filter of 8 Hz but this allowed for intra-step fluctuations to be included in the data and when a lower cutoff was used, agreement with an LPS system substantially improved due to the removal of this part of the signal. What is recommended however, is that the same filter applied to a form of criterion data should also be applied to the dataset being tested. An additional complication is that one filter might work well at improving system agreement at low velocities, but not as well at higher levels (or vice versa), although techniques such as wavelet analysis have been applied to address this (75).

**Figure 6:**
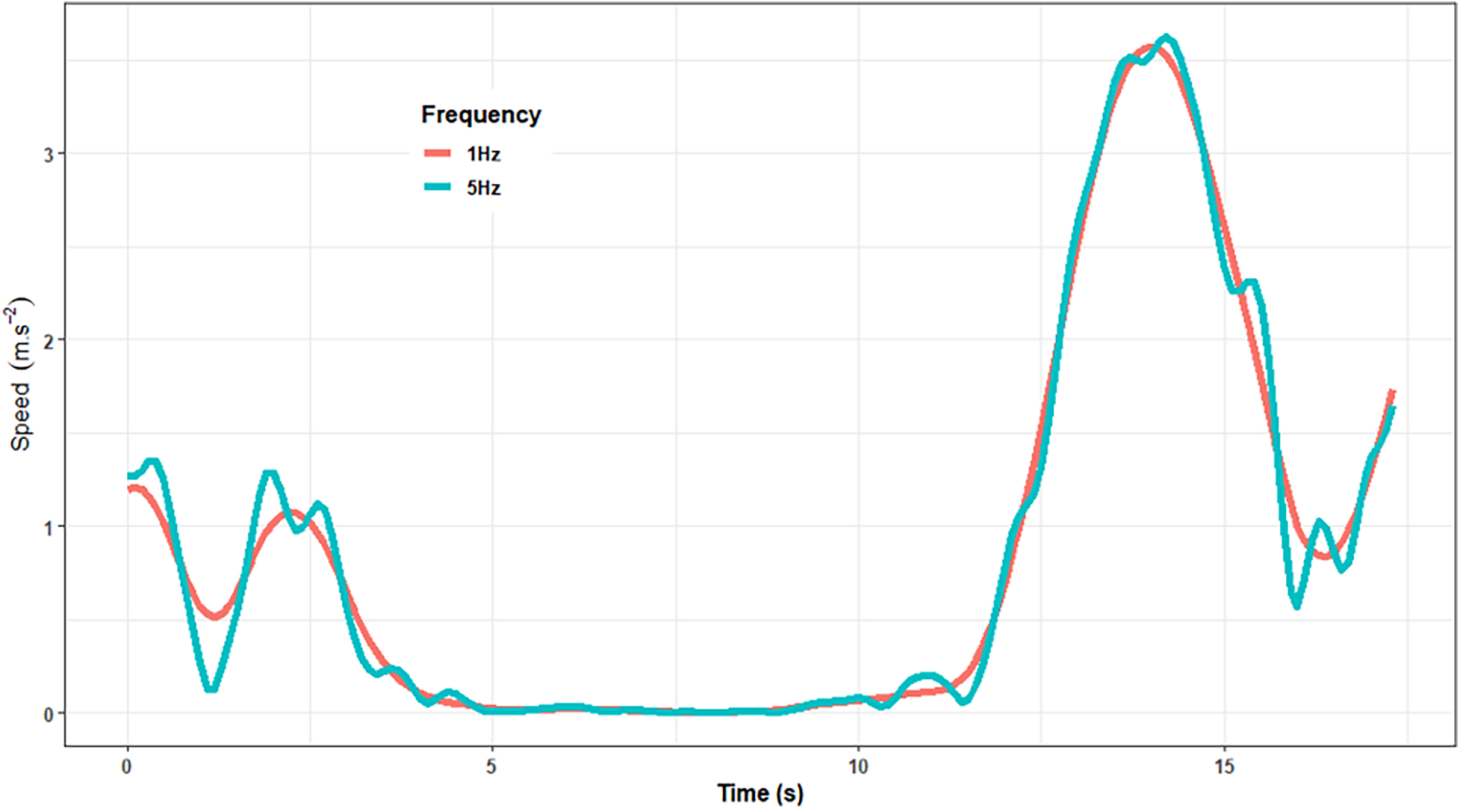
Illustration of the differences exerted by a 1 Hz and 5 Hz filter when applied to a velocity time series. Even a seemingly small consideration such as this can equate to substantially differences being recorded in terms of the distances covered by an athlete across the course of a match or competition.

Figure 6 shows the influence that this seemingly innocuous matter can have on the output of data. Here, a 1 Hz filter applied to a small section of data will clearly influence the way in which a manufacturer compares to a criterion measure. Our analyses show that this has the potential to alter the root mean square difference (RMSD) of a system to a criterion measure from around 0.03 m/s to 0.08 m/s. Whilst this appears negligible, over the course of a 90 min match, it may lead to substantially different inferences being made from two systems in terms of the distance covered by a player.

### Quantification of accuracy & precision

Two predominant considerations exist when considering how to quantify the accuracy and precision of an EPTS. The first relates to the metric(s) extracted from the data. Instantaneous displacement or velocity is typically the most “raw” format along with position (when measured directly rather than derived). However these may be harder to collect by practitioners and more cumbersome to handle comparative to the aggregated values typically delivered to users by EPTS software. For instance, the total distance covered by an athlete in competition may not be as detailed and informative as instantaneous velocity, however it is arguably more easily interpretable by the layperson and easier to communicate to a range of stakeholders. However for the purposes of determining accuracy and precision, the more sophisticated measures should be used as (a) this provides the most detailed investigation into the data and (b) most aggregated measures are derived from these values in any case. The exact level required for each will depend on the nature of the application.

**Figure 7:**
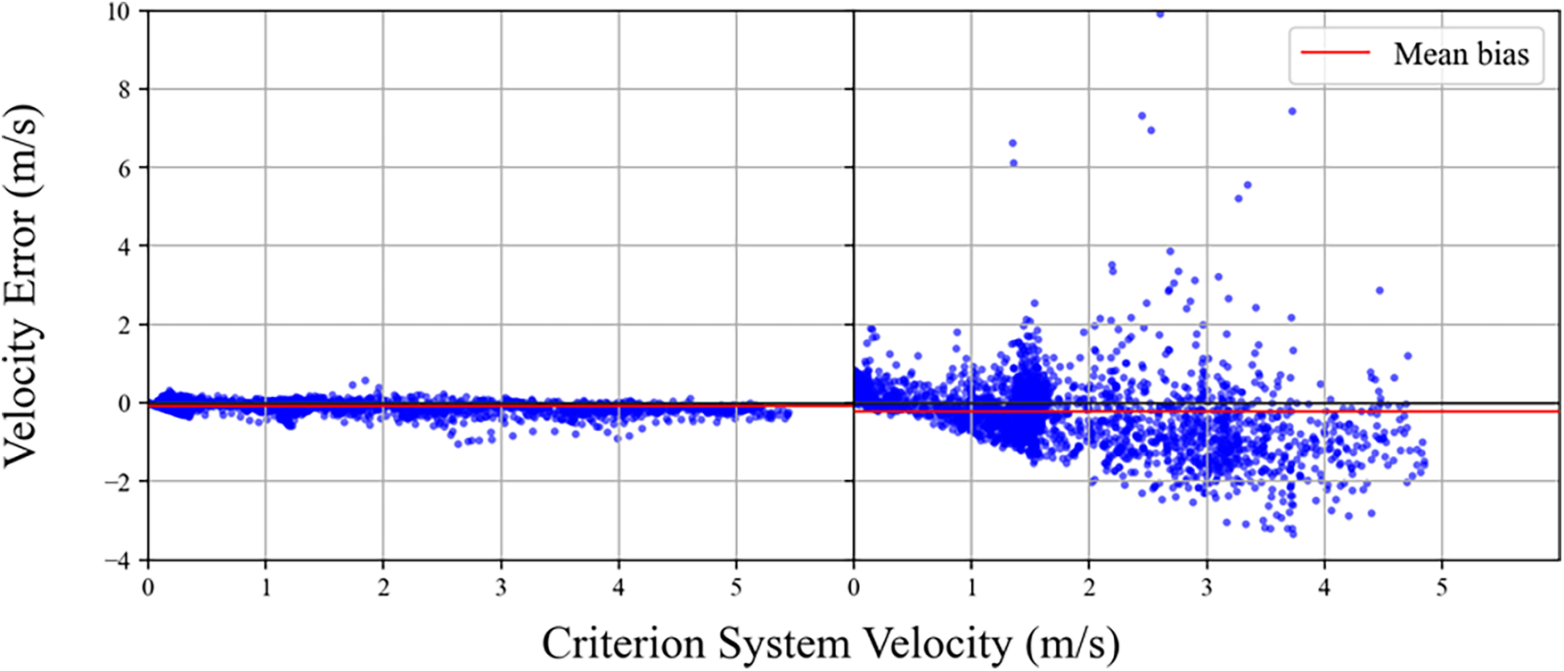
Visual inspection of error in EPTS velocity. Despite the wider range of differences shown on the right hand side of the figure, the mean bias between the two datasets is similar. This highlights the limitations in considering single metrics or visualisations in the assessment of EPTS.

The second consideration relates to the statistical measure(s) used to represent accuracy and precision. Correlational statistics are commonly used and well understood, but have problems when non-linearity in system accuracy is present and may over-estimate performance when the number of samples is high (and viewed in conjunction with statistical significance). These values are also not expressed in relative terms, which somewhat limits their practical utility. Mean bias is often used as a measure of precision to determine the extent to which an EPTS typically will under or over-estimate relative to a concurrent or criterion measure. Figure 7 shows an example of a system whereby as velocity increases, the error remains relatively evenly distributed around zero on the *y* axis. Mean bias may also be insightful when there is a systematic increase in error in one direction (similar to the second plot shown in the figure). A range of issues exist with mean bias however, for example when visually inspected in the plot below shows that the eye is drawn to outliers even though the majority of values are very close to the zero line, thus misrepresenting EPTS performance. Further, when there is substantial error both above and below the zero line on the *y* axis however, the considering the mean bias alone can be misleading as it may record values of close to zero, even though the system is considerably inaccurate.

**Figure 8:**
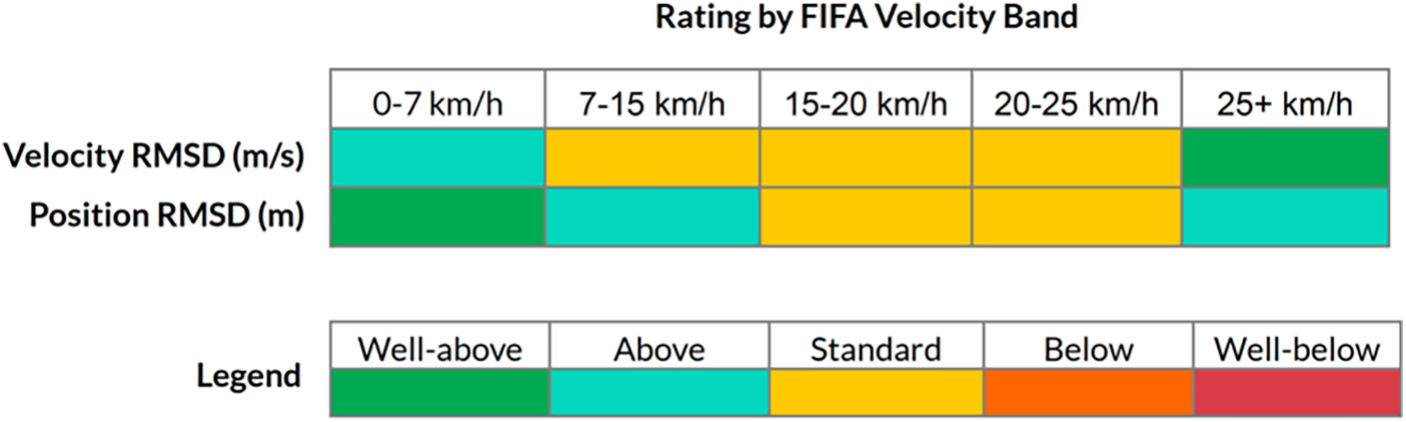
Exemplar report for an EPTS based on the FIFA velocity bands. The rating system implemented by FIFA is shown Available at: https://www.fifa.com/technical/football-technology/standards/epts.

The RMSD works by providing a non-negative value as a representation of accuracy, with readings closer to zero typically better. Similar to mean bias, it doesn't handle non-linearity well however is somewhat sensitive to outliers. Consequently, the two measures are often considered together. When differences between systems are not uniform and are non-linear, it might be useful to express these differences by groupings or bandings. These could be informed by segmenting the error based on “meaningful” changes in errors. However, bandings are commonly used in many sports for prescription and evaluation purposes, based on relative intensities of human movement. Thus establishing a second set of bands for purpose of error/difference distinction would seem impractical. Further, where bands are used, they may differ considerably between different sports or even within sports depending on the league, team or individual involved. Another issue with reporting by velocity bands is that the end user is given the impression that each band should be equally considered in evaluating the overall quality of the system. In Figure 8 below, an exemplar system is provided a rating for each velocity band (based on FIFA's categories) (76).

In most team sports however, athletes spend the majority of time in lower velocity bands. In such scenarios, if higher error values are observed in higher bands but the athlete spends little time reaching those values, it could be argued that is practically acceptable. However, practitioners may argue that these are the very values that need to be measured with high accuracy, thus the importance is greater here. As with many exercises in determining the quality of a system, the level of acceptability may depend on the end user. In the example above for instance, a system that struggles for accuracy at higher velocities may not present a problem when used with younger athletes that don't typically reach those thresholds. Thus, the use case of practitioners will dictate the importance they place on the accuracy of EPTS at varying velocity bands.

**Figure 9:**
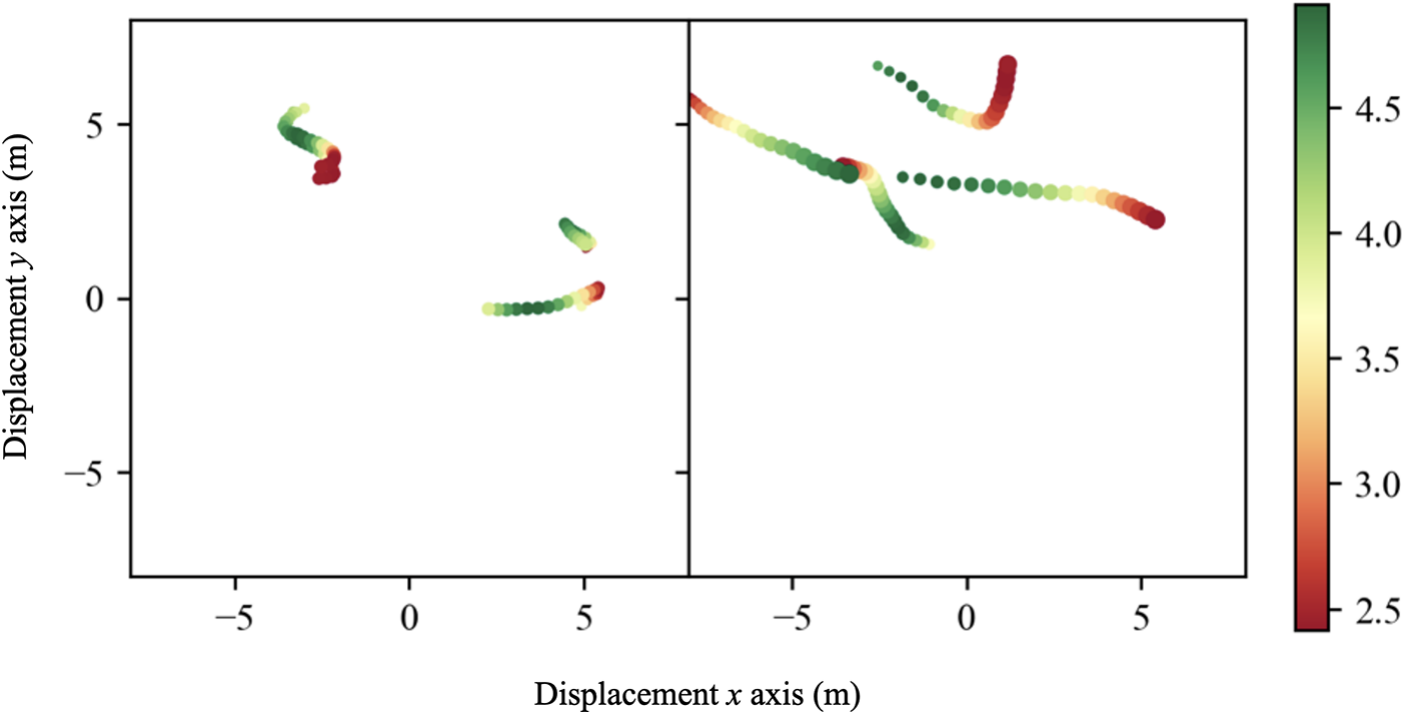
Visualisation of EPTS error. The 99th percentile is visualised along with data frames immediately prior to the movement. A visual representation of the athlete's movement is shown, with their corresponding velocity (m/s) shown using a red to green colour scale.

A raft of work has gone into contextualising athlete and team movement based on features relating to competition itself (i.e., 77, 78). This work has sought to not only consider the influence of velocity magnitude but how contextual information such as pitch location and certain competition events may result in different athlete movement profiles. This same context could also be used to identify those specific movements which create the most difficult conditions for EPTS to track accurately (i.e., occlusion in team sports, unexpected orientation of players, rapid turning movements under deceleration etc). Considering the performance of EPTS in this manner has the advantage of providing manufacturers with research and development feedback as well as providing the user with an understanding of which types of movements should be viewed with caution. An example is shown in Figure 9, whereby the data showing the largest error (the 99th percentile in this instance) has been visualised as part of feedback. Further context could be provided to this (i.e., player identification, position on a field etc).

Given all of these considerations, we recommend that the performance of EPTS should ideally be considered as a range, based on exposure across a range of different conditions, rather than as a single fixed value as is typically reported. For example, an EPTS may be reported based on their typical variation across multiple conditions, such as “if they are playing on a pitch with 4 cameras on elite level footballers, then we expect and RMSD of between 0.15 and 0.30 m/s”. It is suggested that end-users conduct their own investigations into the isolated effect of different factors on differences between system outputs.

## Conclusions

As methods of tracking improve in resolution, regardless of methodology, so to does the opportunity to collect new and higher resolution metrics. These could include the combination of additional sensors but also limb tracking which has become more feasible given the rise of vision-based markerless tracking. This potentially permits new insights into aspects such as gait parameters (79), limb tracking for informing adjudication and even automated classification of events based on spatiotemporal data (80). All of this has the potential to be good news for the end-user. Greater numbers of options means more applications and value however also requires ongoing assessment for quality as per the above. It may also provide greater diversification of product for providers as well.

In summary, data derived from EPTS continues to grow in many sports, with applications across a variety of purposes. In order for end-users to to adopt this data with confidence, they should be aware of the challenges and considerations discussed in this manuscript. This information should also be of use to manufacturers and companies in the EPTS area to help refine current products and guide new research and development projects.
